# Swimming exercise during early-life starvation enhances adult stress resistance in *Caenorhabditis elegans*

**DOI:** 10.17912/micropub.biology.002077

**Published:** 2026-03-30

**Authors:** Natacha S. Zayas-Feliciano, Dinah L. Ramos-Ortolaza

**Affiliations:** 1 Department of Biomedical Sciences, Pontifical Catholic University of Puerto Rico, Ponce, Puerto Rico; 2 Department of Natural Sciences, Pontifical Catholic University of Puerto Rico, Ponce, Puerto Rico

## Abstract

Early-life exposure to adverse conditions, like starvation, can lead to long-term vulnerability to stress. In this study, we used *Caenorhabditis elegans* to evaluate whether swimming exercise during early-life starvation could counteract this detrimental effect. Larvae subjected to swimming while starving showed enhanced resistance to osmotic and heat stress in adulthood. These findings suggest that this type of physical activity during early life stress can promote long-term resistance to environmental challenges. Given that many cellular stress response pathways are conserved across species, this study provides an insight into how early behavioral interventions might enhance stress resilience later in life.

**Figure 1. Effects of swimming on stress resistance in Caenorhabditis elegans:** **(A)** Timeline of experiments for starved animals. Synchronized L1 larvae were transferred to unseeded NGM plates to induce starvation. After 72 hours on starvation, a subset of animals underwent a 15-min swimming session per day for four consecutive days (Starved + Swimming group), while another subset remained on unseeded NGM plates (Starved group). One day after the final swimming session, all animals were transferred to seeded NGM plates to resume normal development at 20 °C. Upon reaching adulthood, they were tested for osmotic and heat stress resistance. **(B)** Timeline of experiments for control (Unstarved) animals. Synchronized L1 larvae were either transferred directly to seeded NGM plates with *Escherichia coli* OP50 (Control group) or subjected to four 15-min swimming sessions on a single day before transfer to seeded plates. All animals were maintained at 20 °C and allowed to develop normally. Upon reaching adulthood, they were tested for osmotic and heat stress resistance. **(C)** Time to paralysis following exposure to 500 mM NaCl was used as a measure of osmotic stress resistance. A significant overall difference was observed between groups, (Kruskal Wallis test, H (3) = 45.23, p < 0.0001). Post hoc Dunn's multiple comparisons test showed that Starved + Swimming animals took significantly longer to paralyze in comparison to Control (p < 0.0001****), Control + Swimming (p = 0.0006***) and Starved animals (p < 0.0001****). No significant differences were found between Control and Control + Swimming (p = 0.1117), Control and Starved (p >&nbsp; 0.9999) or Control + Swimming and Starved (p = 0.4175). **(D)** Percentage of worms that survived a 6-hour heat shock as a measure of heat stress resistance. A significant difference was observed between groups, (Kruskal-Wallis test, H (3) = 22.41, p < 0.0001). Post hoc Dunn's multiple comparisons test showed that a significantly higher percentage of Starved + Swimming animals survived the heat shock in comparison to Starved (p = 0.0005***) and Control animals (p = 0.0153*). A significantly higher percentage of Control + Swimming animals also survived in comparison to Control (p = 0.0472*) and Starved animals (p = 0.0041**). No significant differences were observed between Control and Starved animals (p >&nbsp; 0.9999) or Control + Swimming and Starved + Swimming animals (p >&nbsp; 0.9999).

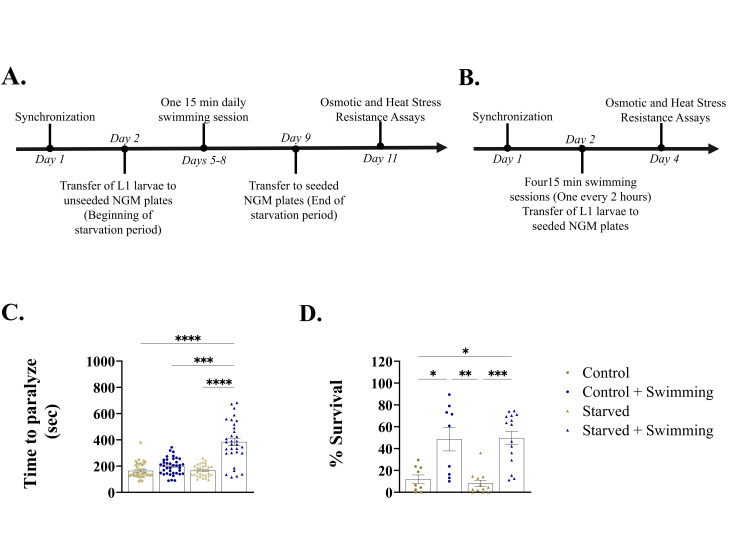



## Description

Early-life stress (ELS) refers to exposure during childhood to adverse physical and/or psychological stimuli to the extent that the individual is unable to adequately cope. It involves a wide range of stressful or traumatic experiences including, but not limited to, malnutrition, abuse, parental loss, and violence. Unfortunately, ELS can induce long-lasting alterations in the developing brain, which can lead to cognitive deficits and an increased lifetime risk of psychiatric disorders, including depression, anxiety and substance abuse (Birnie & Baram, 2025). Although the mechanisms underlying the long-term effects of ELS are not yet fully understood, studies suggest that sustained neurobiological changes may result in altered cognitive function, disrupted reward-processing, and increased sensitivity to stressors (Peña, 2025) further highlighting the potential impact of ELS on brain development and behavior.

Physical activity is a non-pharmacological intervention known to improve stress resilience and reduce symptoms of anxiety and depression (Philippot et al., 2022; Saeed, Cunningham and Bloch, 2019). While these benefits have been documented, less is known about how physical activity influences long-term outcomes when it occurs in the context of early-life adversity. To address this, we used *Caenorhabditis elegans* as a model system in which swimming has been characterized as an exercise paradigm that shares central features of mammalian exercise, including elevated muscle metabolic activity and post-exercise fatigue, as well as altered carbohydrate and lipid metabolism (Laranjeiro et al. 2017). We, therefore, decided to test whether swimming during early-life starvation affects stress resistance in adulthood.

To induce early-life starvation, synchronized *C. elegans* L1 larvae were transferred to unseeded NGM plates. Maintaining animals under these conditions leads to developmental arrest at the L1 larval stage (Jobson et al. 2015), ensuring that animals remain in an early-life developmental state. Three days after the onset of starvation, animals were subjected to a daily 15-minute swimming exercise session for four consecutive days ( Figure 1A ). After each session, they were returned to unseeded NGM plates to maintain starvation conditions. The day after the last swimming session, animals were transferred to seeded NGM plates to resume development. Once they reached adulthood, we assessed osmotic and heat stress resistance.

Animals that were not subjected to starvation (unstarved controls) were maintained on seeded NGM plates throughout the experiment. &nbsp;A subset of these animals underwent four 15-minute swimming exercise sessions, administered at two-hour intervals on the first day after synchronization at the L1 stage. Once they reached adulthood, osmotic and heat stress resistance assays were performed ( Figure 1B ). Although we recognize that the swimming schedules of starved and unstarved control animals may elicit distinct physiological responses, we wanted to ensure that all animals were at the same developmental stage during exercise. Applying the same swimming schedule to both groups would have resulted in unstarved animals undergoing exercise across different developmental stages, introducing greater variability in physiological and behavioral responses.

Our results showed that starved animals that underwent swimming sessions exhibited significantly higher osmotic stress resistance compared to starved animals that did not swim and unstarved controls ( Figure 1C ). In the heat stress assay, animals that underwent swimming sessions showed increased heat stress resistance regardless of whether they had experienced starvation or not ( Figure 1D ). These findings suggest that swimming exercise during early life can enhance stress resistance in adulthood. Although swimming may transiently reduce feeding, resembling a form of dietary restriction, which can enhance stress resistance in *C. elegans* (Greer et al., 2007), our results suggest that this alone does not account for the observed effects. Unstarved animals that underwent swimming and had normal access to food showed an increase in heat stress resistance consistent with the idea that reduced feeding during exercise may partially contribute to this effect. These animals, however, did not exhibit increased resistance to osmotic stress, suggesting that reduced feeding alone may not be sufficient to induce general stress resilience. In contrast, starved animals that underwent swimming exhibited enhanced resistance to both osmotic and heat stress, while starved animals that did not swim showed a significantly lower resistance. These results indicate that swimming during early-live adversity enhances stress resistance possibly through mechanisms that cannot be explained solely by reduced feeding during exercise.

Our findings highlight the potential of physical activity as a protective intervention against the long-term effects of early life adversity. In humans, ELS has been consistently associated with altered stress responsivity, increased vulnerability to psychiatric disorders, and poorer physical health across the lifespan (Heim & Nemeroff, 2001; Levin & Liu, 2021). Strategies that enhance stress resilience, particularly non-pharmacological ones, may offer broad benefits for mental and physical health (Nishimi et al., 2021). In this study, we showed that swimming exercise during early developmental stages increases stress resistance in adult *C. elegans* , including animals not exposed to starvation, suggesting a general benefit of early-life physical activity. While this model does not represent the full complexity of human psychological or physiological stress, many cellular and molecular stress response pathways are conserved. Our results, therefore, provide a foundation for investigating how early behavioral interventions, such as physical activity, may promote long-term resilience to environmental challenges. Future studies should explore the mechanisms underlying these effects and examine whether similar benefits extend across different stressors and developmental stages.

## Methods

**Strains:** *Caenorhabditis elegans* N2 strain was obtained from the *Caenorhabditis* Genetics Center at the University of Minnesota. Worms were cultured at 20 °C on Nematode Growth Medium (NGM) seeded with a streptomycin resistant strain of *Escherichia coli* ( OP50 ), as a food source.

**Synchronization:** To obtain a homogeneous population of worms, we performed a synchronization protocol based on that from Porta-de-la-Riva et al. (2012). Briefly, gravid adults were transferred to 2.0 ml tubes and washed three times with M9 buffer. They were then treated with a 4% Sodium hypochlorite solution. Obtained eggs were washed three times with M9 buffer and kept rotating overnight at room temperature for hatching. L1 larvae were then transferred to NGM plates and kept at 20 °C unless otherwise specified.

**Starvation** : Synchronized L1 larvae were maintained at 20 °C in unseeded NGM plates for 7 days to induce L1 arrest and starvation as an early life stressor (Jobson et al. 2015).

**Physical activity:** Worms underwent swimming exercise following a protocol adapted from Schmidt et al (2021). In each session, worms were transferred to 35 mm unseeded NGM plates containing 1 ml of M9 buffer and allowed to swim for 15 minutes. During this time, plates were left undisturbed on the benchtop and covered with a cardboard box to minimize external stimuli that could affect movement, and to block light exposure. After each session, worms were collected, returned to their respective NGM plates, and maintained at 20 °C.

Two distinct groups were subjected to the swimming protocol. The first group experienced early-life stress as previously described. Worms from this group, which were developmentally arrested at the L1 stage, underwent one daily swimming session for four consecutive days, starting two days after synchronization. The second group, referred to as the Control + Swimming group, was not subjected to starvation, therefore progressed through normal development. To ensure that these worms were still in the larval stage during the swimming sessions, they underwent four swimming sessions spaced two hours apart, conducted one day after synchronization.

**Osmotic Stress Resistance Assay:** Upon reaching adulthood, between 100-150 worms were collected and placed at the center of a 35 mm NGM plate containing 500 mM NaCl, according to the protocol described by Naβ et al. (2021). A stopwatch was started immediately to record the time required for all animals to completely paralyze. We performed six independent trials, with 6-9 plates per group per trial.

**Heat Stress Resistance Assay:** Upon reaching adulthood, worms were collected and subjected to heat shock by incubating at 37 °C for 6 hours, following the protocol described by Naβ et al. (2021). After the heat shock, worms returned to 20 °C. After 20 hours, survival was assessed by gently prodding the worms with a platinum wire. Worms that failed to respond were scored as dead. Heat stress resistance was assessed in five independent trials with 3-6 plates per group per trial. In some trials, plates with overcrowded animals were excluded from the analysis. An average of 740 animals per group was analyzed across the trials. Where feasible, experimenters were blinded to treatment during scoring of both stress resistance assays.

**Statistical Analysis:** All statistical analysis were performed using GraphPad Prism (Version 10.6.1). Data were first assessed for normality using the Shapiro-Wilk test. In both the heat and osmotic stress resistance experiments, several groups violated normality assumptions. To account for this, the non-parametric tests Kruskal-Wallis test was performed, followed by Dunn's multiple comparisons test.

## Reagents

**Table:** 

**Strain**	**Genotype**	**Available from**
N2	*C. elegans* wild isolate	CGC
*Escherichia coli*	OP50-1 – Streptomycin resistant	CGC
